# Characteristics of SARS-CoV-2 Transmission among Meat Processing Workers in Nebraska, USA, and Effectiveness of Risk Mitigation Measures

**DOI:** 10.3201/eid2704.204800

**Published:** 2021-04

**Authors:** Jocelyn J. Herstein, Abraham Degarege, Derry Stover, Christopher Austin, Michelle M. Schwedhelm, James V. Lawler, John J. Lowe, Athena K. Ramos, Matthew Donahue

**Affiliations:** University of Nebraska Medical Center, Omaha, Nebraska, USA (J.J. Herstein, A. Degarege, J.V. Lawler, J.J. Lowe, A.K. Ramos);; Nebraska Department of Health and Human Services, Lincoln, Nebraska, USA (D. Stover, C. Austin, M. Donahue);; Nebraska Medicine, Omaha (M.M. Schwedhelm, J.V. Lawler)

**Keywords:** coronavirus disease, COVID-19, infection prevention, masks, meat processing industries, occupational exposures, physical barriers, policy interventions, SARS-CoV-2, viruses, severe acute respiratory syndrome coronavirus

## Abstract

The coronavirus disease (COVID-19) pandemic has severely impacted the meat processing industry in the United States. We sought to detail demographics and outcomes of severe acute respiratory syndrome coronavirus 2 infections among workers in Nebraska meat processing facilities and determine the effects of initiating universal mask policies and installing physical barriers at 13 meat processing facilities. During April 1–July 31, 2020, COVID-19 was diagnosed in 5,002 Nebraska meat processing workers (attack rate 19%). After initiating both universal masking and physical barrier interventions, 8/13 facilities showed a statistically significant reduction in COVID-19 incidence in <10 days. Characteristics and incidence of confirmed cases aligned with many nationwide trends becoming apparent during this pandemic: specifically, high attack rates among meat processing industry workers, disproportionately high risk of adverse outcomes among ethnic and racial minority groups and men, and effectiveness of using multiple prevention and control interventions to reduce disease transmission.

In the early months of the coronavirus disease (COVID-19) pandemic, meat processing facilities became among the largest epicenters of COVID-19 outbreaks in the United States (*1*). Declared a critical infrastructure industry in April 2020 (*2*), meat processing facilities are particularly vulnerable to COVID-19 because of the high density of workers required for operations, prolonged close contact of personnel on the production line, indoor work environments with compact cafeteria and locker room areas, and a workforce with diverse cultural and linguistic backgrounds that make educational efforts more challenging (*3*). A Centers for Disease Control and Prevention (CDC) report found that, as of May 31, 2020, >16,000 workers in meat and poultry processing facilities in the United States had been diagnosed with COVID-19 and 86 had died (*4*); as of October 2020, those case counts and deaths had more than tripled (*5*). 

Meat processing facilities in Nebraska employ ≈26,000 workers (*6*). The first COVID-19 illness among meat processing facility workers in Nebraska was identified March 9, 2020. As of July 2020, cases had been reported among workers in 23 Nebraska meat processing facilities. The University of Nebraska Medical Center (UNMC) and Nebraska Department of Health and Human Services partnered to mitigate COVID-19 risks in Nebraska among workers in this industry. Nebraska Department of Health and Human Services expanded case investigations and contact tracing teams and coordinated 2 mass testing events with participating meat processing facilities. UNMC created evidence-based guidelines for facilities (*7*) and assembled a team of infectious disease and infection prevention and control (IPC) experts to provide onsite and virtual technical assistance to facilities to evaluate gaps in IPC practices and provide facility-specific IPC recommendations. 

Local and state health departments conducted case investigations to collect information on demographics, employer, occupation, industry, illness descriptions, medical history, and outcomes among Nebraska meat processing workers. Moreover, although industry-specific guidelines for mitigating COVID-19 transmission in meat processing facilities have been issued by CDC and other public health organizations (*7*,*8*), the effectiveness of these measures among workers has not been reported. We present data on the effectiveness of initiating a universal mask policy and installing physical barriers (plexiglass or plastic partitions) between workstations and at cafeteria tables on reducing COVID-19 incidence at meat processing facilities in Nebraska. 

## Methods 

### Characteristics of Laboratory-Confirmed Cases

We used SAS version 9.4 (https://www.sas.com) to develop a keyword algorithm to identify meat processing facility workers by using occupation, industry, and employer data fields from case investigations conducted among Nebraska residents with laboratory-confirmed COVID-19. Specimens were collected from healthcare providers, work-sponsored testing events, state-sponsored testing events, and stationary state-sponsored testing sites during April 1–July 31, 2020. We used R 4.0.2 with dplyr version 1.0.2 (https://cran.r-project.org) to examine the duration of timelines between illness onset dates, specimen collection dates, and case investigation dates. Data from records with erroneous timelines were excluded from timeline analyses (Table 1), including records in which the same dates were recorded for illness onset, specimen collection, and investigation (not possible within the case investigation workflow) or if illness onset occurred after the case investigation. 

**Table 1:** Timeline exclusions, subsets, and median durations for case investigations of severe acute respiratory syndrome coronavirus 2 infection among meat processing workers, Nebraska, April 1–July 31, 2020*

Category	Illness onset to specimen collection	Specimen collection to Investigation	Illness onset to investigation
Beginning total	3,695	4,834	3,817
Erroneous timelines excluded: onset date = collection date = investigation date	16	16	16
Erroneous timelines excluded: investigation → onset	39	39	40
Probable cases analyzed separately: onset = investigation → collection	29	29	31
Probable cases analyzed separately: onset → collection = investigation	76	116	76
Probable cases analyzed separately: onset → investigation → collection	19	55	19
Presymptomatic cases analyzed separately: collection → onset → investigation	214	214	214
Primary timeline totals: onset → collection → investigation	3,302	4,365	3,421
Median (range) for primary timeline, d	3 (0–53)	4 (1–109)	8 (1–110)

We classified workers with laboratory-confirmed COVID-19 into 1 of 3 timeline categories based on the relationship between the illness onset date, specimen collection date, and case investigation date: primary timeline, probable timeline, or presymptomatic timeline. We classified workers into the primary timeline if they had an illness onset date followed by specimen collection date, followed by investigation date. We classified workers into the probable timeline if they were investigated before their positive test result was available. We further classified workers in the probable timeline into 3 subcategories: 1) illness onset occurring the same day as the investigation date, followed by specimen collection; 2) illness onset date followed by an investigation date, followed by specimen collection date; or 3) illness onset date followed by specimen collection date occurring on the same day the investigation began. We classified workers into the presymptomatic timeline if they had a specimen collection date followed by illness onset date, followed by an investigation date. We used the R package table1 version 1.2 to create frequency tables for demographics, illness descriptions, medical history, and outcomes.

### Effects of Mask and Physical Barrier Interventions 

The UNMC team provided technical assistance as voluntarily requested by meat processing facilities in the state to identify facility-specific recommendations on additional risk mitigation measures that could be implemented. We used a 4-page checklist summarizing primary IPC recommendations for meat processing facilities to guide technical assistance site visits or calls and subsequent debriefing with plant leadership (*7*). The checklist included recommendations for engineering controls (e.g., enhancing ventilation, installing physical barriers between workers on the production line and in cafeterias), administrative controls (e.g., cohorting of consistent work teams, education, environmental cleaning and disinfection policies), and personal protective equipment. Site visit personnel completed the checklist and gathered information on the workforce (e.g., number of employees, employee demographics) and dates of initiating a universal mask policy, installing physical barriers, or both. For each facility, the dates of initiating a universal mask policy and completing physical barrier installation were collated and used in the analyses. 

We used Stata version 16 (https://www.stata.com) to conduct a retrospective analysis of data on the incidence of severe acute respiratory syndrome coronavirus 2 (SARS-CoV-2) infection among employees in the meat processing facilities that received technical assistance during April–July 2020. To estimate when the effect of an intervention, measured by case counts, might be observed, we estimated the total duration from exposure through testing and diagnosis (positive test) at ≈10 days based on prior analyses (*9*). The main outcome variable we assessed was the incidence of SARS-CoV-2 infection. 

For each plant, before-intervention incidence per 1,000 persons per day was calculated by dividing the number of cases reported before or <10 days after intervention by the product of the total number of employees and the number of days from baseline to 10 days after intervention. Postintervention incidence per 1,000 persons per day was calculated by dividing the number of new cases reported 10 days after intervention by the product of the total number of cases 10 days after intervention and the number of days from 10 days after intervention to the last day of the study period. Z-test of proportion was used to compare incidence per 1,000 persons per day before or <10 days after intervention with incidence 10 days after the intervention. Differences in the incidence of SARS-CoV-2 infection before and after the intervention were considered significant where p was <0.05. 

## Results 

### Characteristics of Laboratory-Confirmed and Probable Cases

During April 1–July 31, 2020, among Nebraska residents working at meat processing facilities, 5,002 of ≈26,000 received a diagnosis of COVID-19 (attack rate 19%). Of those, 3,817 (76%) had a recorded illness onset date, 4,834 (97%) had a recorded specimen collection date, and 5,002 (100%) had a recorded investigation date; 3,695 (74%) had both illness onset and specimen collection dates recorded, 4,834 (97%) had both specimen collection and investigation dates recorded, and 3,817 (76%) had both illness onset and investigation dates recorded. After excluding erroneous timelines, probable cases, and presymptomatic cases (Table 1), we used data from confirmed cases with illness onset followed by specimen collection followed by investigation to calculate durations for the primary timeline; we calculated durations independently for the probable and presymptomatic timelines.

For the primary timeline, the median duration from illness onset to specimen collection was 3 days (n = 3,302), from specimen collection to investigation was 4 days (n = 4,365), and from illness onset to investigation was 8 days (n = 3,421). For probable cases, the median duration from illness onset to specimen collection was 4 days (n = 124), from specimen collection to investigation was 0 days (n = 200), and from illness onset to investigation was 2.5 days (n = 124). For presymptomatic cases, median duration from specimen collection to illness onset was 3 days (n = 214), from specimen collection to investigation was 6 days (n = 214), and from illness onset to investigation was 4 days (n = 214). 

Among the 5,002 total COVID-19 case-patients, the median age was 43 years (mean 42.7 years, range 13–81 years). Men accounted for 2,919 (58%) cases; 3,343 (67%) identified as Hispanic or Latino, 2,678 (54%) as White, 570 (11%) as Asian, 405 (8%) as Black or African American, 27 (<1%) as American Indian or Alaska Native, 16 (<1%) as Native Hawaiian or other Pacific Islander, and 1,306 (26%) as other race or unknown. Twenty-seven case-patients (<1%) were pregnant. 

Symptoms were reported by 4,237 (85%) workers, 501 (10%) were asymptomatic, and 264 (5%) had unknown symptom status. Of those reporting symptoms, the average illness duration was 12.8 days (median 11 days). Headache (2,526; 60%), cough (2,442; 58%), and muscle pain (2,344; 55%) were most frequently reported symptoms. Smoking (124/4,237; 2%) was reported more frequently among workers not identifying as Hispanic or Latino (64; 5%) than among Hispanic or Latino workers (52; 2%). A preexisting medical condition was reported by 1,117 (22%) workers, most frequently diabetes (359; 32%) or cardiovascular disease (240; 21%). Diabetes was reported more frequently among workers identifying as Hispanic or Latino (277; 36%) than among non-Hispanic/Latino workers (72; 23%). 

Among symptomatic case-patients, 225 (4%; median age 55 years, range 19–49 years) were hospitalized for an average duration of 8.4 days and 83 (2%; median age 57 years, range 21–79 years) required intensive care unit (ICU) admission; 21 (<1%; median age 63 years, range 39–79 years) workers died. Among the 225 hospitalized patients, 161 (72%) were men, as were 65 (78%) requiring ICU admission, and 17 (81%) who died. Hispanic or Latino ethnicity was reported for 164 (73%) hospitalized patients, 65 (78%) requiring ICU admission, and 18 (86%) who died. 

### Effects of Mask and Physical Barrier Interventions

We analyzed case counts and intervention initiation dates for 13 facilities for which data were available; technical assistance was provided onsite at 12 facilities and by telephone call to 1 facility. Facilities consisted of primary processing plants for beef (n = 7), pork (n = 3), and poultry (n = 1), as well as 2 secondary processing plants. The number of workers employed at the 13 facilities ranged from <400 to several thousand (mean 1,675). Placement of physical barriers varied by facility, but they were generally located on the production line and at cafeteria tables; barriers consisted of plexiglass partitions, plastic wrap secured around PVC pipes, or plastic sheeting. Although the site visit teams recommended use of surgical masks, national shortages of personal protective equipment early in the pandemic led to the adoption of different masking requirements; some facilities allowed cloth masks, and other facilities acquired and provided surgical masks to workers. Of the 13 facilities, 5 (38%) initiated a universal mask policy >10 days before physical barriers were installed; 6 (46%) initiated a universal mask policy and installed physical barriers <10 days apart; and 2 (15%) had universal mask policies but no physical barriers in place at the time of technical assistance and whether physical barriers were installed later is unknown. 

We analyzed the incidence of SARS-CoV-2 infection before and after the date the last intervention was initiated (e.g., date physical barriers were installed if universal mask policy began first). Ten days after the last intervention was initiated, 8 facilities (62%) showed a statistically significant (p<0.05) decrease in incidence and 3 (23%) showed a nonsignificant decrease; 1 (7%) facility showed a statistically significant (p<0.05) increase in incidence and 1 (7%) showed a nonsignificant increase in incidence (Table 2). Three facilities reported case counts from the time between initiating mask policy and physical barrier interventions that allowed us to compare incidence before mask intervention, between mask and physical barrier initiation, and after both were in place simultaneously (Table 3). All 3 facilities showed a significant reduction (p<0.05) in incidence, particularly with both interventions deployed. 

**Table 2:** Comparisons of the incidence of severe acute respiratory syndrome coronavirus 2 infection before and after mask or physical barrier interventions or both among employees in 13 meatpacking facilities in Nebraska, April–July 2020*

Facility	Incidence /1,000 persons /d		
	<10 d after final intervention	10 d after final intervention	p value for difference
Facilities that initiated a universal mask policy >10 d before physical barriers			
A	7.27	0.33	<0.001
B	3.21	0.69	<0.001
C	3.46	0.27	<0.001
D	3.64	0.15	0.072
E	0.48	2.09	0.008
Facilities that initiated a universal mask policy and physical barriers <10 d of each other			
F	17.16	0.58	<0.001
G	2.49	1.27	0.002
H	4.08	0.78	<0.001
I	6.82	1.40	<0.001
J	2.19	0.059	<0.001
K	0.65	1.90	0.180
Facilities that only initiated a universal mask policy			
L	3.2	2.87	0.745
M	3.29	3.178	0.944
*For facilities that initiated both a universal mask policy and physical barriers, date of last intervention was defined as start date of latter intervention (i.e., if physical barriers were initiated first, final intervention date was date of mask policy initiation). For facilities that initiated only masking, final intervention date was the initiation date.			

**Table 3:** Comparisons of the incidence of severe acute respiratory syndrome coronavirus 2 infection among meat processing workers before mask intervention, between mask and physical barrier intervention, and after physical barrier intervention in meatpacking facilities, Nebraska, April–July 2020

Facility	Incidence /1,000 persons /d			
	<10 d after mask intervention	Between day 10 after mask and day 10 after physical barrier intervention	>10 d after physical intervention	p value for difference*
A	3.46	3.23	0.26	<0.001
B	11.13	42.2	0.58	<0.001
C	2.63	0.26	0.32	<0.001
*p value difference represents difference in incidence before initiation of mask intervention and after physical barrier intervention.				

## Discussion

The meat processing industry in Nebraska employs ≈26,000 workers (*6*), of whom 5,002 were diagnosed with COVID-19 during March–July 2020. The attack rate during this time period (19%) was more than double the 9.1% attack rate that was reported in a multistate analysis of meat processing facilities across the United States through May 2020 (*4*). Cases in meat processing facilities have far-reaching effects, potentially fueling outbreaks within surrounding communities where workers and workers’ families comprise a substantial proportion of area residents. In addition, plants are often located in rural communities with limited infrastructure and resources to respond to outbreaks. In Nebraska, the 8 counties with the highest COVID-19 case rates per capita (as of September 2020) are also home to large meat processing facilities (*10*). 

This report supports the increasing body of evidence that the COVID-19 pandemic has disproportionately affected racial and ethnic minority groups (*11*). Although 67% of confirmed cases were among Nebraska meat processing workers reporting Hispanic or Latino ethnicity, they constituted 73% of hospitalized case-patients, 78% of ICU admissions, and 86% of deaths, indicating a higher proportion of poor outcomes (hospitalizations, ICU admissions, deaths) compared with other racial and ethnic groups. Likewise, data presented here reflect emerging evidence suggesting disease manifestations are more severe in men (*12*,*13*). Despite comprising 58% of confirmed case-patients, men represented 72% of hospitalized case-patients, 78% of ICU admissions, and 81% of deaths. Higher risk of poor outcomes among men and ethnic and racial minority groups demands tailored prevention and education strategies to subgroups shown to be more affected by adverse outcomes, both for this specific work environment and for broader local, state, and federal public health policy applications. Plant or corporate management can work to address these disparities among their worker populations by engaging with language and culture experts to ensure appropriate and effective communication and educational materials (e.g., videos, infographics) by providing materials in all languages spoken by workers and partnering with respected local community leaders (e.g., religious and spiritual leaders, elders) and community organizations to educate and disseminate information to workers. 

The proportion of SARS-CoV-2 infections that remain asymptomatic is uncertain, although some reports estimate it to be higher than 30% (*14*–*16*). The percentage of asymptomatic COVID-19 cases (10%) among Nebraska meat processing workers was lower than these estimates. However, because few Nebraska meat processing facilities completed mass testing events during the study period, it is likely that many asymptomatic cases went undetected and are not reflected in this report. Indeed, a mass testing event at 1 Nebraska facility found that nearly one third of workers confirmed with COVID-19 reported no symptoms (*17*). Data on the 214 presymptomatic case-patients described in this report suggest detectable levels of virus in these persons and therefore transmission potential (*18*–*20*) at a median of 3 days before onset. Mass and routine testing enables identification of asymptomatic and presymptomatic infections, leading to swifter isolation, fewer days of potential exposure, and faster identification, quarantine, and testing of close contacts. As detailed in this report, identifying presymptomatic cases shortened the duration from symptom onset to investigation by a median of 4 days. Facilitywide or corporationwide routine testing programs, with frequency of testing informed by both local community transmission rates and cases identified within the plant, can position meat processing plants to identify cases early and stem potential outbreaks.

Risk mitigation strategies based on symptoms, such as active screening protocols and paid sick leave policies, are limited by asymptomatic and presymptomatic transmission and emphasize the importance of multilayered IPC interventions. Industry-specific guidance released by the CDC and the Occupational Safety and Health Administration in late April (*8*) centered on the Hierarchy of Controls risk mitigation framework (https://www.cdc.gov/niosh/topics/hierarchy/default.html) to reduce transmission within facilities (*21*). Although many of these risk mitigation strategies are similar to those recommended for various other high-risk industries (e.g., schools, long-term care facilities), the effectiveness of these IPC measures in the meat processing work environment has not been reported. 

Our results indicate significantly reduced incidence of COVID-19 cases in 62% of studied facilities following adoption of universal masking and physical barrier interventions. Several factors may explain why some facilities did not see incidence decrease and 1 saw incidence significantly increase after initiating these measures. First, as an engineering control, physical barriers are generally considered one of the most effective measures to reduce person-to-person transmission of a communicable disease because they do not rely on worker adherence (*21*). However, since the study period, evidence has mounted supporting the substantial role of aerosols in transmitting COVID-19 (*22*–*24*). Although physical barriers installed between meat processing workers on the production line and at cafeteria tables would block larger respiratory droplets, the primary mode of transmission according to the CDC (*22*), they would not fully protect against aerosol transmission. Moreover, low temperatures and limited fresh air supply combined with physically demanding work conditions could facilitate longer-range aerosol transmission (*25*). Enhancements in ventilation (e.g., increasing the number of air exchanges per hour, installing high efficiency particulate air [HEPA] filtration) should therefore be considered the most effective engineering control for COVID-19. More study is needed on aerosol transmission dynamics in this setting.

Second, although masking is one of the most effective tools for reducing COVID-19 transmission (*26*,*27*), the effectiveness of a universal mask policy relies on workers being educated on and adhering to proper mask use. A previous study of Nebraska meat processing workers found that only 44% of workers had received information on how to wear and care for a mask properly (*28*). Observed adherence to proper mask use (e.g., wearing the mask over both mouth and nose, minimizing adjustment or touching of the front of the mask) varied during site visits; at some plants, nearly all workers exhibited proper use, but at other facilities nearly half of workers wore masks below their noses. 

The IPC challenges inherent in meat processing facilities cannot be addressed with only 1 or 2 measures; multilayered interventions are more effective than any single measure (*29*). In addition to IPC-focused strategies to reduce transmission within the facility, such as reducing density, engineering controls, physical distancing, active screening, environmental cleaning and disinfection, and masking, workforce policies ensuring social protections such as paid sick leave and flexible absenteeism policies are critical tools to prevent the disease from entering the workplace. However, given the inherent IPC challenges faced by the industry (e.g., high density of workers, duration of shifts, indoor environment, crowded cafeterias where masks are removed), it is also possible that no combination of interventions will be completely effective at reducing transmission in meat processing facilities, particularly when high rates of local community transmission exist. Facilities that did not see a significant reduction in incidence after initiating mask policies and physical barriers may not have incorporated other strategies to the same degree as facilities that did see significantly reduced incidence. Alternatively, some facilities we assessed might have initiated key interventions well before cases among their workers were diagnosed, causing interventions to appear less effective in this study. 

A limitation of this study is that, although we attempted to distinguish the effectiveness of a universal mask policy from that of physical barrier installation, only 3 facilities had enough cases between the initiation of the 2 interventions to evaluate the separate direct effects of the measures. Moreover, when it became apparent in mid-April that meat processing facilities were particularly vulnerable to and being affected by COVID-19, facilities scrambled to incorporate IPC strategies within a short timeline and requested simultaneous technical assistance from our team. In many cases, site visits were conducted <10 days after a universal mask policy or physical barrier installation was begun. Our site visits and incorporation of additional IPC measures beyond physical barriers and masking might have contributed to reduced incidence. In addition, we were not able to definitively separate out whether transmission to case-patients occurred in the workplace or in the community and therefore couldn’t determine the exact effect risk mitigation measures had on incidence compared with trends in community transmission rates. However, COVID-19 cases among meat processing workers represented almost 1 in 5 cases in Nebraska during the study period (there were 27,036 total cases in Nebraska from the beginning of the pandemic through July 31, 2020) (*30*). In addition, Nebraska’s first wave of COVID-19 cases peaked in early May and gradually declined from May to July; our findings indicate that mitigation measures had a more rapid effect on incidence than reductions reflected in community transmission trends. 

In conclusion, we present a snapshot of the effect of COVID-19 among meat processing workers in facilities in Nebraska. Nearly 1 in 5 Nebraska meat processing workers were diagnosed with COVID-19 between March and July 2020, a profound burden of cases unparalleled in any other worker population. Many of the nationwide trends that have become apparent during this pandemic applied here, namely high attack rates among workers in the meat processing industry, a disproportionately high risk of adverse outcomes among ethnic and racial minority groups and men, and the effectiveness of IPC interventions at reducing person-to-person transmission. Increased multilayered IPC strategies, rapid contact tracing, and accessible testing are critical to identifying asymptomatic and presymptomatic cases and interrupting silent transmission. COVID-19 will be an enduring threat to the meat processing industry and its workers for the foreseeable future. Facilities must adopt and sustain multiple interventions to prevent, control, and rapidly identify transmission within facilities to protect this worker population.
